# Supplementary material for: Hearing as adaptive cascaded envelope interpolation
Source: Commun Biol. 2023 Jun 24;6:671. doi: 10.1038/s42003-023-05040-5 (PMC10290642; doi:10.1038/s42003-023-05040-5)
Supplement: Supplementary file 2 — Supplementary Information [file 42003_2023_5040_MOESM2_ESM.pdf]

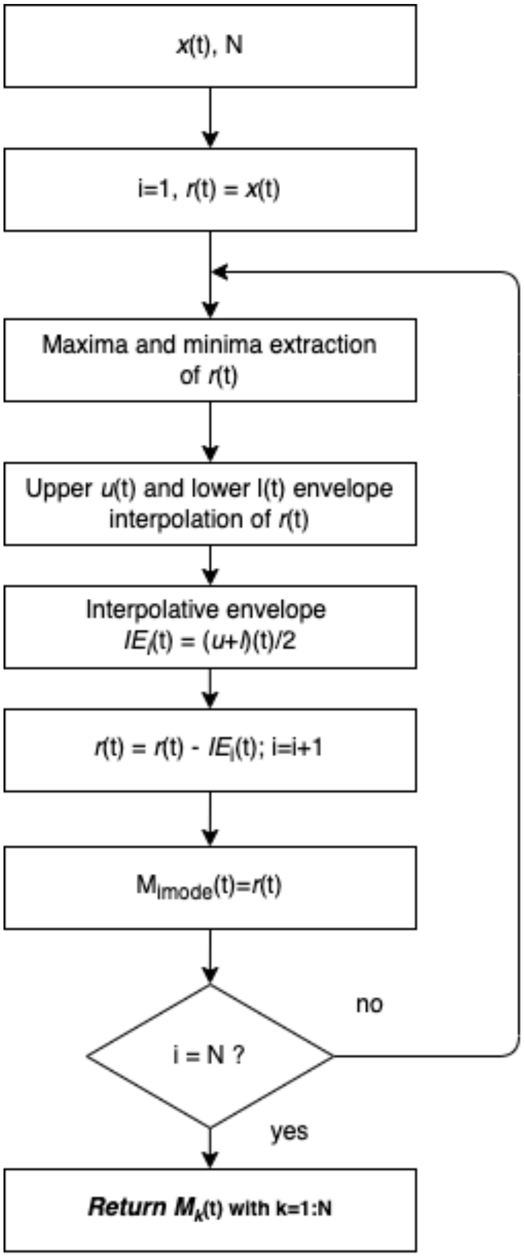

Supplementary Figure 1. Summary of the different steps of the Cascaded Envelope Interpolation (CEI). CEI is largely inspired by the Empirical Mode Decomposition (EMD) approach. CEI and EMD differ by the number of iterations: CEI uses only one iteration and a fixed number of modes N, while the number of iterations and the number of modes in EMD depends on the signal. The maxima and minima of the signal are firstly extracted on the signal s(t). From this set of extrema, the upper and lower envelopes are then obtained through interpolation by cubic splines and then averaged together to form the interpolative envelope (IE) which is thereby subtracted from the signal s(t) to compute the first mode M_1 of the decomposition which is the residual r(t). The interpolative envelope is then passed through the same process in order to compute the second mode, the process is repeated N times, 6 in the paper.

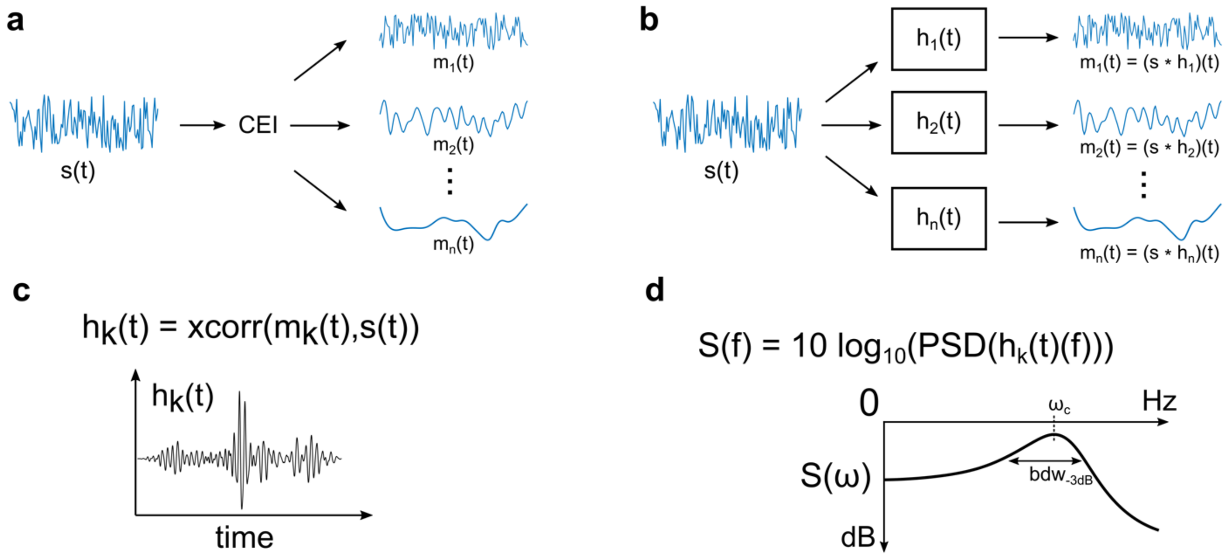

Supplementary Figure 2. CEI as an adaptive filter-bank. The goal of this analysis is to evaluate whether the equivalent linear filtering operated by CEI is adaptive, meaning that it naturally adapts their spectral shapes to the spectral content of the analyzed sounds. In particular, we aim to investigate whether the properties, i.e., the relationship between bandwidth and center frequency, conforms or not with the known Equivalent Rectangular Bandwidth (ERB) model. The speech and environmental sounds are 1500 excerpts from the Making Sense of Sounds (MSoS) challenge taken from the Freesound database (Font, Roma & Serra, 2013), the ESC-50 dataset (Piczak, 2015). We chunked the 1500 excerpts in segments of 800ms leading to 148500 short sound excerpts. **a.** For each excerpt s(t), we computed 6 modes $m_i(t)$ with CEI, with *i* the mode number. **b.** Each mode was considered as the result of a convolution between the initial signal s(t) and an impulse response $h_i(t)$. **c.** The equivalent impulse response $h_k(t)$ was computed using a cross-correlation (xcorr) between the mode $m_k(t)$ and the original signal s(t), with k the mode number. **d.** Each filter was analyzed based on a model of its spectral shape $S(f) = 10\, log_{10}(PSD(h_k(t))(f))$, where PSD is the Power Spectrum Density of the impulse response $h_k(t)$, and f is the frequency in Hz. We assume that each filter is roughly a band pass, and we hereby determine its center frequency f_c and bandwidth bdw_{-3dB}.

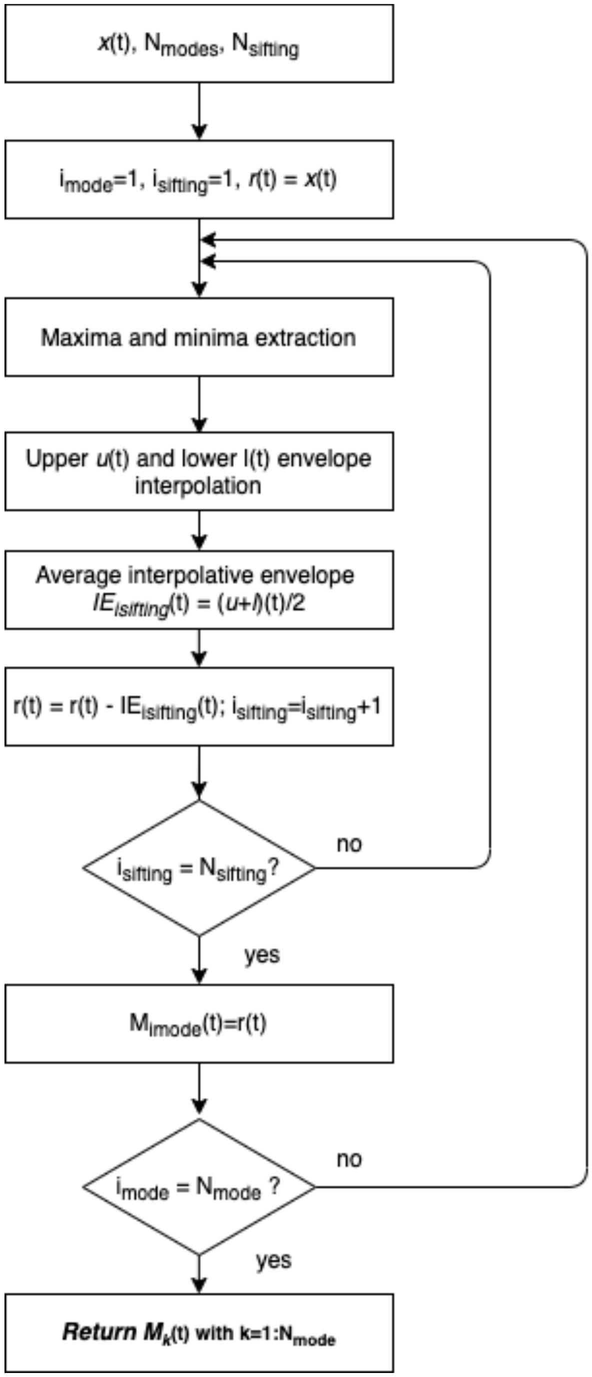

Supplementary Figure 3. Summary of the different steps of the Empirical Mode Decomposition. Empirical Mode Decomposition is the generalization of the CEI process with an iterative process, called sifting which is imbricated in each mode extraction. In EMD, this process is repeated iteratively, the envelope interpolation and subtraction, until the interpolative envelope reaches a criterion of monotony. As such a threshold process is not biologically plausible as the number of needed iterations is not known. We therefore propose an intermediate approach with the same sifting process as EMD, but with a fixed number of iterations defined a priori. CEI consists of this pipeline with only 1 sifting iteration.

Supplementary References

Font, F., Roma, G., & Serra, X. (2013, October). Freesound technical demo. In Proceedings of the 21st ACM international conference on Multimedia (pp. 411-412).

Piczak, K. J. (2015, October). ESC: Dataset for environmental sound classification. In Proceedings of the 23rd ACM international conference on Multimedia (pp. 1015-1018).
